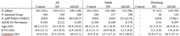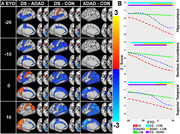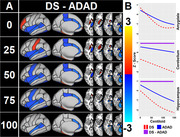# Brain Volumetric Trajectories in Down Syndrome and Autosomal Dominant Alzheimer Disease

**DOI:** 10.1002/alz.089313

**Published:** 2025-01-09

**Authors:** James Tyler Kennedy, Julie K. Wisch, Brian A. Gordon, Anna H. Boerwinkle, Tammie L.S. Benzinger, William E Klunk, Michael Rafii, Sid E. O'Bryant, Julie C Price, Michael A. Yassa, Mithra Sathishkumar, Liv McMillan, Adam M. Brickman, Patrick J. Lao, Charles M Laymon, Sharon J. Krinsky‐McHale, Florence Lai, H. Diana Rosas, Sigan L Hartley, Shahid Zaman, Ira T. Lott, Joseph H. Lee, Ricardo Allegri, Sarah Berman, Jasmeer P. Chhatwal, Helena C Chui, Carlos Cruchaga, Martin R. Farlow, Gregory S Day, Jae‐Hong Lee, Johannes Levin, Ralph N Martins, Hiroshi Mori, Richard J. Perrin, Stephen Salloway, Raquel Sanchez‐Valle, Peter W. Schofield, Chengjie Xiong, Jason J. Hassenstab, Eric McDade, Randall J. Bateman, Benjamin L Handen, Elizabeth Head, Nicole Schupf, Mark Mapstone, Bradley T. Christian, Beau Ances, Dominantly Inherited Alzheimer Network and the Alzheimer's Biomarker Consortium‐Down Syndrome

**Affiliations:** ^1^ Washington University in St. Louis School of Medicine, St. Louis, MO USA; ^2^ Department of Radiology, Washington University School of Medicine, Saint Louis, MO USA; ^3^ Washington University in St. Louis, St. Louis, MO USA; ^4^ University of Pittsburgh, Pittsburgh, PA USA; ^5^ Alzheimer's Therapeutic Research Institute, San Diego, CA USA; ^6^ University of North Texas Health Science Center, Fort Worth, TX USA; ^7^ Massachusetts General Hospital, Boston, MA USA; ^8^ University of California, Irvine, Irvine, CA USA; ^9^ Columbia University, New York, NY USA; ^10^ Columbia University Irving Medical Center, New York, NY USA; ^11^ Department of Radiology and Bioengineering, University of Pittsburgh, Pittsburgh, PA USA; ^12^ New York State Institute for Basic Research in Developmental Disabilities, Staten Island, NY USA; ^13^ Harvard/Massachusetts General Hospital, Boston, MA USA; ^14^ University of Wisconsin‐Madison, Madison, WI USA; ^15^ Cambridge Intellectual and Developmental Disabilities Research Group, Department of Psychiatry, University of Cambridge, Douglas House, Cambridge UK; ^16^ Taub Institute for Research on Alzheimer’s Disease and the Aging Brain, Columbia University, New York, NY USA; ^17^ INEBA, Buenos Aires Argentina; ^18^ Brigham and Women's Hospital, Boston, MA USA; ^19^ Department of Neurology, Keck School of Medicine, University of Southern California, Los Angeles, CA USA; ^20^ Washington University School of Medicine, Saint Louis, MO USA; ^21^ Indiana Alzheimer's Disease Research Center, Indianapolis, IN USA; ^22^ Mayo Clinic, Jacksonville, FL USA; ^23^ Department of Neurology, Asan Medical Center, University of Ulsan College of Medicine, Seoul Korea, Republic of (South); ^24^ German Center for Neurodegenerative Diseases (DZNE), Munich Germany; ^25^ Edith Cowan University, Perth, Western Australia Australia; ^26^ Osaka City University Medical School, Osaka Japan; ^27^ Alpert Medical School, Brown University, Providence, RI USA; ^28^ Alzheimer’s disease and other cognitive disorders Unit. Hospital Clínic de Barcelona; FRCB‐IDIBAPS; University of Barcelona, Barcelona Spain; ^29^ University of Newcastle, Newcastle, NSW Australia; ^30^ The Charles F. and Joanne Knight Alzheimer Disease Research Center, St. Louis, MO USA; ^31^ Washington University St. Louis, St. Louis, MO USA; ^32^ Washington University in St. Louis, School of Medicine, St. Louis, MO USA; ^33^ The UC Irvine Institute for Memory Impairments and Neurological Disorders (UCI MIND), Irvine, CA USA

## Abstract

**Background:**

Alzheimer disease (AD) related cognitive decline occurs at relatively young ages in individuals with Down syndrome (DS, early‐mid 50s) and in those with autosomal dominant mutations (ADAD, 40‐50s). Both groups show similar patterns of amyloid accumulation. We examined if brain volumes are similarly affected by AD pathology in individuals with DS and ADAD.

**Method:**

Data for cognitively stable and declining participants was obtained from the Alzheimer Biomarker Consortium‐Down Syndrome (ABC‐DS) and the Dominantly Inherited Alzheimer Network (DIAN). Stability/decline was identified based on cognitive testing and interview of individuals and caregivers by trained assessors. Cognitively stable family members without DS/ADAD mutations were recruited as controls from both studies. Participants underwent MRI and amyloid positron emission tomography (PET) scans from which brain volumes and amyloid (centiloids) were derived, respectively. Participants from DIAN had Pittsburgh Compound‐B (PIB) scans, ABC‐DS had PIB or florbetapir. Nonlinear cross‐sectional associations between regional brain volumes and estimated years to onset of cognitive decline (EYO, negative values before onset, positive after) and centiloid were evaluated using generalized additive models while controlling for sex and random effects of family. EYO was set to 52 for all participants with DS and based on parental decline/mutation type for participants with ADAD. EYO for controls was based on the EYO of their family member.

**Result:**

Data from 239 participants with DS (47 declining), and 340 participants with ADAD (122 declining), and 263 familial controls were included. Higher EYO and centiloid values were associated with lower brain volumes in almost all regions. At earlier EYOs, individuals with DS typically had smaller regional volumes than ADAD or sibling controls, with volume declining linearly across the EYO range. By contrast, ADAD mutation carriers had similar volumes to non‐carriers at early EYOs, with volumes diverging as early as 10 years before decline. Brain volumes and centiloid values were inversely related in ADAD and DSAD. Volume in key cortical regions were similar by the expected year of onset in both groups.

**Conclusion:**

ADAD and DSAD demonstrated different temporal patterns of regional neurodegeneration prior to cognitive change despite being similarly affected by early onset amyloid.